# A large non-parasitic population of *Savalia savaglia* (Bertoloni, 1819) in the Boka Kotorska Bay (Montenegro)

**DOI:** 10.1038/s41598-024-58101-y

**Published:** 2024-04-02

**Authors:** Martina Canessa, Egidio Trainito, Giorgio Bavestrello, Slavica Petović, Nikola Đorđević, Vesna Mačić

**Affiliations:** 1 Dipartimento Di Scienze Della Terra Dell’Ambiente E Della Vita (DISTAV), Università Di Genova, Corso Europa, 26 -16132 Genova, Italy; 2 NBFC, National Biodiversity Future Center, Palermo, Italy; 3 Genoa Marine Centre-Stazione Zoologica Anton Dohrn, Ecologia E Biotecnologie Marine, Istituto Nazionale di Biologia, Villa del Principe, Piazza del Principe, 4 - 16126 Genova, Italy; 4 Consorzio Nazionale Interuniversitario Per Le Scienze del Mare, Piazzale Flaminio 9, 00196 Roma, Italy; 5 Institute of Marine Biology, University of Montenegro, Put I Bokeljske Brigade 68, 85330 Kotor, Montenegro

**Keywords:** Parazoanthidae, Infralittoral zone, *Vrulja*, Asexual reproduction, Animal forests, Marine protected areas, Mediterranean Sea, Adriatic Sea, Biodiversity, Zoology

## Abstract

The golden coral *Savalia savaglia* is a long-living ecosystem engineer of Mediterranean circalittoral assemblages, able to induce necrosis of gorgonians’ and black corals’ coenenchyme and grow on their cleaned organic skeleton. Despite its rarity, in Boka Kotorska Bay (Montenegro) a shallow population of more than 1000 colonies was recorded close to underwater freshwater springs, which create very peculiar environmental conditions. In this context, the species was extremely abundant at two sites, while gorgonians were rare. The abundance and size of *S. savaglia* colonies and the diversity of the entire benthic assemblage were investigated by photographic sampling in a depth range of 0–35 m. Several living fragments of *S. savaglia* spread on the sea floor and small settled colonies (< 5 cm high) suggested a high incidence of asexual reproduction and a non-parasitic behaviour of this population. This was confirmed by studying thin sections of the basal portion of the trunk where the central core, generally represented by the remains of the gorgonian host skeleton, was lacking. The *S. savaglia* population of Boka Kotorska Bay forms the unique Mediterranean assemblage of the species deserving the definition of animal forest. Recently, temporary mitigation measures for anthropogenic impact were issued by the Government of Montenegro. Nevertheless, due to the importance of the sites the establishment of a permanent Marine Protected Area is strongly recommended.

## Introduction

The golden coral *Savalia savaglia* (Bertoloni, 1819) is the unique Mediterranean zoanthid able to deposit its own organic skeleton and the parasitic aptitude to induce necrosis in gorgonians (e.g., *Eunicella* spp., *Leptogorgia* spp. and *Paramuricea* spp.) and anthipatarians and progressively overgrow their cleaned axis^1–7^. The species is particularly prone to be damaged by fishing activities^8,9^, so much so that it appears in Annex II of Specially Protected Areas of Mediterranean Importance (SPAMI-Barcelona Convention), Appendix II of the Berne Convention and is classified as Near Threatened in the International Union for Conservation of Nature (IUCN) Red List since 2015^10^.

In 1819, the species was formally described as *Gorgonia savaglia* by the botanist Antonio Bertoloni using as type specimen a colony present in the collection of Francesco Ginanni coming from Ragusa (today Dubrovnik), on the eastern coast of the Adriatic Sea^11^. Before Bertoloni’s description, the species was already known for the Adriatic Sea and named “*La Savaglia*” by Ginanni (in Bertoloni^12^) and Donati (in Nardo^13^). The first record of this species (as *Gerardia lamarki*) in the western Mediterranean was due to Lacaze-Duthiers^14^ who produced a very detailed description of specimens collected between Cape Bon (Tunisia) and La Calle (now El Kala, Algeria).

In 1958, at Punta Manara (Ligurian Sea, Italy), the first North Mediterranean specimens were recorded during a diving survey by the pioneer of scientific diving Gianni Roghi^11,15^. In the following years, diving activity and, more recently, Remote Operative Vehicle (ROV) prospections demonstrated the wide distribution of this species in the western and eastern Mediterranean basins^16^. Today, the occurrence of the species has been assessed in the whole Mediterranean Sea and on the Atlantic coast of Portugal, Spain, the Canary Archipelago and Madeira^7,17–21^. Most recently the species was recorded along the French Basque coast of the Bay of Biscay, with colonies presumably established on *Paramuricea grayi* (Johnson, 1861) at 35–40 m depth^22^.

In its entire geographic distribution, the species is still considered rare^2^, in agreement with its parasitic habitus. The only place where the species forms a forest with more than 300 colonies is the Boka Kotorska Bay, in Montenegro^7^. In 2021, on the basis of these records and studies from different projects^23–26^, the Government of Montenegro^27^ has issued measures of protection for two areas (Sopot and Dražin vrt, of 2.9 and 1.2 ha, respectively), where navigation, anchoring and fishing activities are now banned. These areas, showing the highest density of *S. savaglia,* correspond to the location of large karstic underwater springs (*vrulja*) that, during Winter and Spring, produce an outgoing flow of freshwater visible from the surface^28^. In the same localities, huge amounts of freshwater also flow from the reliefs above, mainly from two streams. The first diving exploration of the area observed that the release of cold freshwater, the mixing of water masses and the consequent turbidity of water, have favoured the growth of a *S. savaglia* forest, with colonies exceeding one meter in height^29^.

Unfortunately, until today, a detailed characterization of the benthic assemblage present in these two areas, and in particular of the *S. savaglia* population, remains lacking. The aim of this study is to describe this assemblage and, in particular, the golden coral population living in a unique environmental frame^20^.

## Materials and methods

### Study area

**Figure 1:**
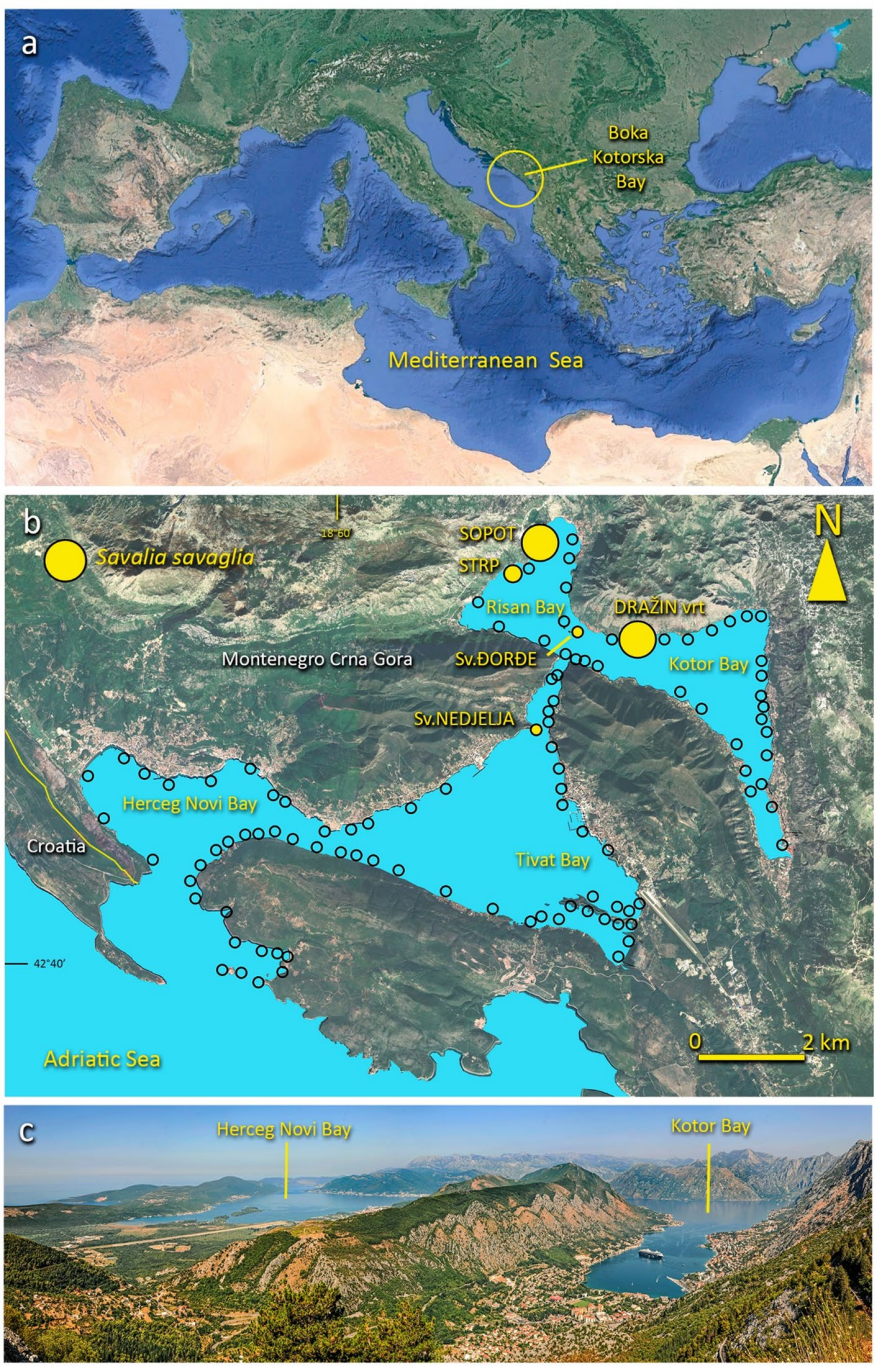
(**a**,**b**) Location of the Boka Kotorska Bay and (**c**) panoramic view of the bay. The occurrence of *Savalia savaglia* (yellow dots) (**b**) is limited to only five sites, located in the inner part of the bay. In contrast, in the other localities (empty dots) no colonies were found. Image under Public Domain—all rights released by Wikipedia: https://it.wikipedia.org/wiki/Mar_Mediterraneo#/media/File:Mediterranean_Sea_16.61811E_38.99124N.jpg. The Boka Kotorska Bay image is modified by Google Earth—Landsat/Copernicus.

Boka Kotorska Bay is considered the largest bay in the Adriatic Sea, often described as the “European southernmost fjord” because of the steep and high slopes that surround it, and it is, in fact, a river valley drowned by tectonic movement^30,31^. The bay, with a surface of 87.3 km^2^ and a coastal perimeter of 105.7 km, is divided into 4 smaller inlets (Fig. 1). The outermost ones, Herceg Novi and Tivat Bays, through the Strait of Verige (only 350 m wide), are connected to the inner part, Risan and Kotor Bays. In Boka Kotorska, precipitations reach the maximum in Europe (4500 mm per year^32^), with high variations throughout the year and almost no rainfall in late Spring and Summer. A huge amount of freshwater flows into the bay from five small rivers, and also from numerous streams and karstic underwater springs—locally called *vrulja*—that are more common in the inner part of the bay (Kotor and Risan Bays). This is particularly evident in the site of Sopot, where a large intermittent spring^28^ at about 30 m depth produces an outgoing water flow visible from the surface, that in April is estimated in 15–20 m^3^s^−1^, while in August it presents a reduced flow, not visible from the surface^29^. This freshwater input and meteorological conditions mainly drive water circulation with high values of residence times in the inner part of the bay^33^. This fact determines a peculiar hydrographic situation, strongly influencing the biological community^23,29,34^.

The seafloor is mostly covered by well-stratified sedimentary layers of fine mud and sand^35^. Rocky bottom and bioconstructions are concentrated in the inner part of the bay^25,36^.

In recent years, a very rapid increase in coastal urbanization and cruise ship traffic has determined a strong anthropogenic pressure on the bay habitats^37^. However two sites hosting the *S. savaglia* forests in the inner part of the Boka Kotorska Bay, Sopot and Dražin vrt, (Fig. 1), have been placed under preventive protection by the decision of the Government of Montenegro in July 2021 and delimited with buoy fences.

### Data collection

In this paper we have summarized unpublished data on the occurrence of *S. savaglia* along the whole coast of the bay (Fig. 1); moreover, the benthic communities of the two main sites including *S. savaglia* forests of Sopot in Risan Bay and Dražin vrt in Kotor Bay were investigated by 20 SCUBA dives in August 2023 in the depth range 0–30 m.

Data on seawater temperature and salinity were obtained by HOBO data loggers (Onset Computers Corporation) placed in the main *S. savaglia* patches at Dražin vrt and Sopot (at 16 and 14 m depth respectively) from May 2018 to December 2020 and additionally only for Dražin vrt from November 2022 to November 2023.

At each site, delimited by buoys anchored on the seabottom, the multi-zoom photographic approach^38^ was used to characterize the benthic assemblages, and in particular, the presence of erected species, and the occurrence of marine litter. For each site about 1100 images were randomly taken covering an average surface of 0.5 m^2^ each (Table 1). Images were captured using a Sony A6000 camera (24 megapixels, two Inon S2000 strobes, color temperature 5000 K) with Sony 16–50 lens (focal length 19 mm), Nauticam WW1 wet wide lens (130° rectilinear field angle) and a Sea & Sea MDX-A6000 underwater case with a flat porthole. Panoramic renderings of the sites to localize the colonies were created with multiple shots subsequently joined and optimized in postproduction using the Photoshop CS6 Merge tool^39^. Photographs were analysed to identify species to the lowest possible taxonomic level. All the colonies of *S. savaglia* present in the two delimited areas were counted. The real density was difficult to estimate because the zoanthids were arranged in dense and crowded patches. The count of colonies is the result of an estimation that considered each identifiable fan as a colony, being impossible to verify if the bases were separated and being the colonies frequently anastomosed on each other.

**Table 1:** Location, depth range, specific richness and abundance of *Savalia savaglia* and density of marine litter found at two investigated sites of the Boka Kotorska Bay.

Site	Coordinates		Depth range (m)	N photos	N species	*S. savaglia* colonies (N)	Marine litter (objects m^-2^)
	Lat (E)	Long (N)					
Dražin vrt	18°42′54’’	42°28′59’’	0–28	1100	63	∼ 509	0.18
Sopot	18°40′48’’	42°30′37’’	0–24	1100	67	∼ 683	0.14

On the base of the photographs, the presence of epibiosis, necrosis and evidence of mechanical damages and/or entanglement on the colonies were recorded.

In the aim to study morphometric features, 75 isolated colonies of the zoanthid were photographed together with a rule for each site. In these images, the height, basal diameter and number of apexes of the colonies were obtained using ImageJ software^40^ and the correlations between colony height, number of apexes and basal diameter were obtained to describe the growth pattern of the species. Moreover, the fractal dimension (FD) was evaluated according to the “box-counting method”, randomly placing photographs of the zoanthid under six 256- mm side grids partitioned into squares along each edge, with n varying from 5 to 50. For each image, the log–log plot of the number of squares entered by the outline of the colony against the number of squares along one side of the grid was obtained through linear regression analysis: the slope of the resulting line equals the FD^41,42^.

To verify the putative parasitic behavior of the species (i.e. presence of the skeleton of gorgonian host) five samples of basal trunks, from 3 mm to 4 cm in diameter, were collected from living and dead colonies in Dražin vrt. These basal portions were embedded in epossidic resin, cut with a diamond saw, mounted on a glass slide and then ground smooth using progressively finer abrasive grit until a thickness of 30 μm. The structure of the organic skeleton was also studied after maceration in NaOH 2M at 45 °C for 5 days.

## Results

### Water temperature and salinity

**Figure 2:**
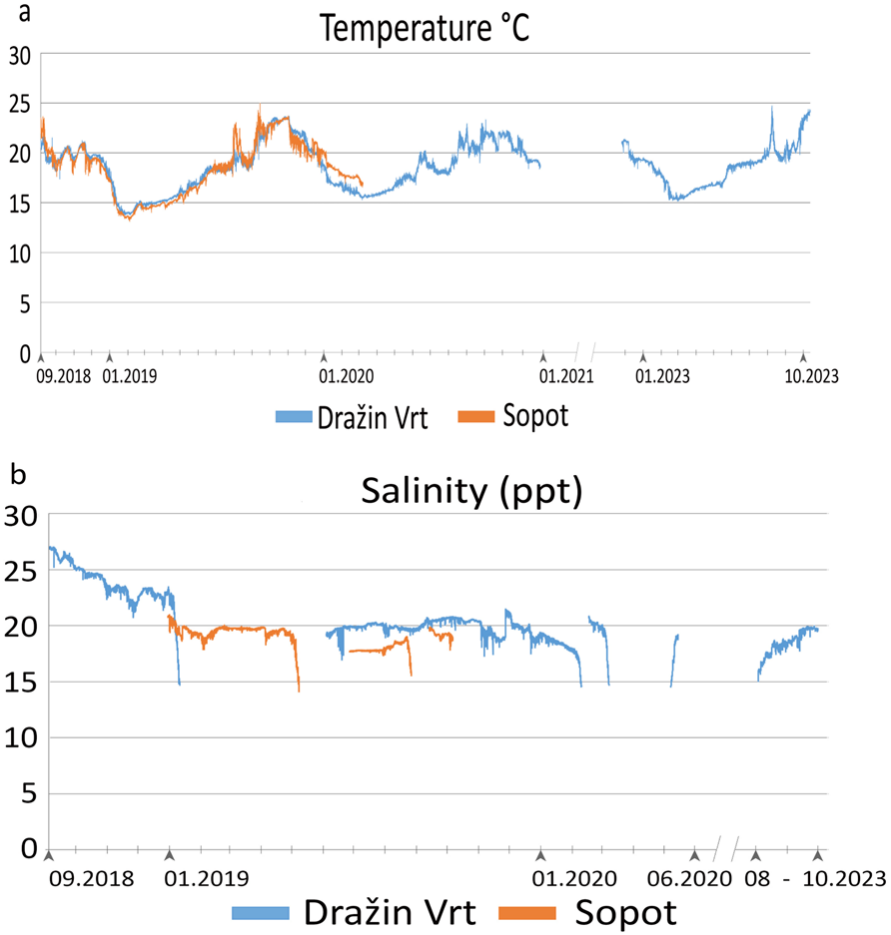
Temperature (**a**) and salinity (**b**) trends concerning the *vrulja* activity, showing the very wide range of values and oscillations recorded during the years. Measurement was recorded inside the main *Savalia savaglia* patches. Blu lines, Dražin vrt; orange line, Sopot.

The two studied locations are very similar in terms of environmental parameters. Data series obtained for temperature inside the main *Savalia savaglia* patches are almost the same for both locations (Fig. 2). Temperature variation has been from 13.8 °C to 24.7 °C during almost 3 calendar years (2019, 2020 and 2023) at Dražin vrt and from 13.1 °C to 24.9 ° C at Sopot, during almost two years (2019 and 2020). The highest peak of temperature was recorded in August 2023 and interestingly similar in October, which was also the case at the end of summer 2019, but for 1–2 °C less (Fig. 2a). Further monitoring will show if the heat waves in 2023 made higher consequences to the anthozoan populations. Variations of salinity were much higher (Fig. 2b) because of the *vrulja*—submarine freshwater springs—and configuration of the surrounding terrain or better to say inflow of rainwater. During many periods of the monitored years data were missing because of two different reasons. One part of the problem was technical and corresponds to the low sensibility of data loggers in an environment of very low salinity, especially in periods when the decrease of salinity is very fast (in relation to heavy rains). Another part of the problem was epiphytes that sometimes were overgrowing data loggers interfering operation of the probes. Based on the available data we can say that salinity never has been more than 27 ppt and mostly was indicating brackish water, 18–22 ppt. Unfortunately, this is not enough for an appropriate representation of the environment, because the data loggers were not measuring under 15ppt and usually were stopped because of very fast changing values of salinity during many periods of monitored years.

### The benthic communities of Boka Kotorska Bay

**Figure 3:**
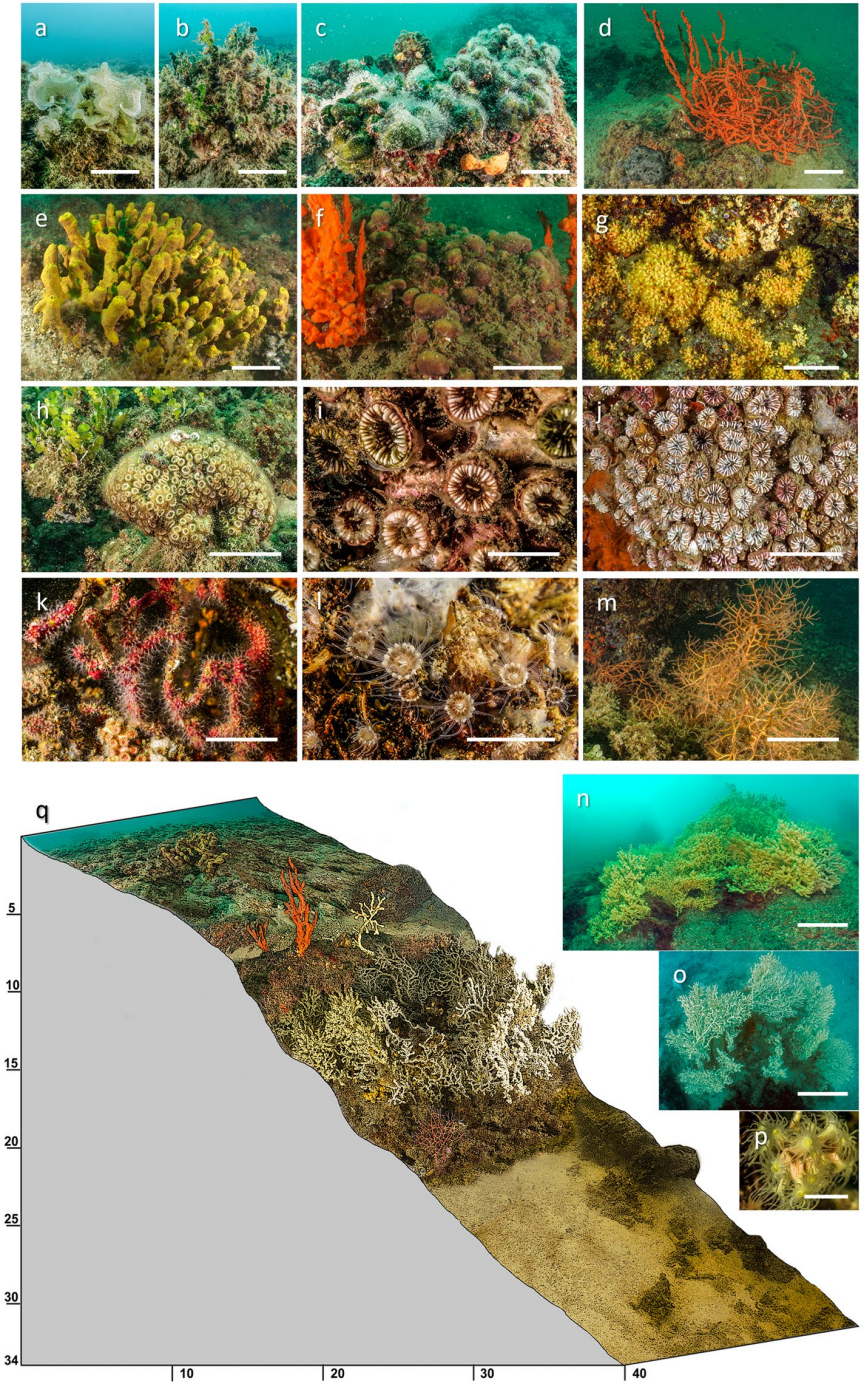
The benthic assemblages found in the Boka Kotorska Bay, where *Savalia savaglia* settled are mainly characterized by the emischiphilous algae *Padina pavonica* (**a**) *Halimeda tuna* (**b**) and *Codium effusum* (**c**); the sponges *Axinella cannabina* (d) *Aplysina* sp. (e) and *Chondrilla nucula* (**f**); the anthozoans *Parazoanthus axinellae* (**g**) *Cladocora caespitosa* (**h**) *Polycyathus muellerae* (**i**) *Phyllangia americana mouchezii* (**j**) *Sarcodictyon catenatum* (**k**) *Epizoanthus* sp. (**l**) and *Leptogorgia sarmentosa* (**m**) *S. savaglia* forests (**n**–**p**). (**q**) Schematic view of the bathymetric distribution of the main benthic species in the studied localities. The letters on the scheme correspond to those of the photos. Scale bars: (**a**–**h**,**k**,**m**–**o**) 10 cm; (**i**,**j**,**l**,**p**) 1 cm.

At the two investigated sites 87 species of sessile benthic organisms were found, with 67 and 63 species recorded at Dražin vrt and Sopot respectively: the main taxonomic animal groups were sponges, (35 species; 40.22%), and anthozoans (12 species; 13.79%). One-half of the species (44) were common for both sites (Table 2). Within the ubiquitarians species, the main group was constituted by algae (mainly *Codium effusum* (Rafinesque) Delle Chiaje, 1829, *Dyctiota dichotoma* (Hudson) Lamouroux, 1809, *Padina pavonica* (Linnaeus) Thivy, 1960, *Halimeda tuna* (Ellis & Solander) Lamouroux, 1816, *Peyssonnelia* spp.) (18.39%), massive sponges as *Aplysina* sp*.*, *Axinella cannabina* (Esper, 1794), *A. polypoides* Schmidt, 1862, *Chondrilla nucula* Schmidt, 1862*, Cymbaxinella* spp., *Dysidea avara* (Schmidt, 1862), *Sarcotragus* spp., anthozoans mainly the zoanthids *S. savaglia*, *Epizoanthus* sp. and *Parazoanthus axinellae* (Schmidt, 1862), the scleractinians *Cladocora caespitosa* (Linnaeus, 1767), *Phyllangia americana mouchezii* (Lacaze-Duthiers, 1897) and *Polycyathus muellerae* (Abel, 1959), the octocorals *Leptogorgia sarmentosa* (Esper, 1791), *Spinimuricea clavareni* (Carpine & Grasshoff, 1975) and *Sarcodictyon catenatum* Forbes in Johnston, 1847 (Fig. 3). The remaining number of found species (27.61%) was composed of mollusks, serpulids, bryozoans, echinoderms and ascidians (Table 2).

**Table 2:** List of species of the benthic communities at the two explored sites.

Species	Dražin vrt	Sopot	Species	Dražin vrt	Sopot
Algae			Anthozoans		
*Asparagopsis taxiformis*	x	–	*Alcyonium coralloides*	x	–
*Codium bursa*	x	–	*Alicia mirabilis*	–	x
*Codium effusum*	x	x	*Caryophyllia inornata*	x	–
Corallinales encrusting	x	x	*Cladocora caespitosa*	x	x
*Cystoseira corniculata*	x	x	*Epizoanthus* sp.	–	x
*Dictyota dichotoma*	x	x	*Leptogorgia sarmentosa*	x	X
*Flabellia petiolata*	x	x	*Madracis pharensis*	x	–
*Halimeda tuna*	x	x	*Parazoanthus axinellae*	x	x
*Padina pavonica*	x	x	*Phyllangia americana mouchezii*	x	x
*Peyssonnelia* spp.	x	x	*Polycyathus muellerae*	x	x
*Peyssonnelia squamaria*	x	x	*Sarcodictyon catenatum*	x	–
*Peyssonelia rubra*	x	x	*Savalia savaglia*	x	x
*Pseudochlorodesmis furcellata*	x	x	*Spinimuricea klavereni*	x	–
*Sargassum* sp.	–	x	Bryozoans		
*Tricleocarpa fragilis*	x	–	*Frondipora verrucosa*	–	x
*Womersleyella setacea*	x	x	*Reteporella grimaldii*	x	–
Sponges			*Schizomavella cf. mamillata*	x	x
*Acanthella acuta*	x	x	Molluscs		
*Agelas oroides*	x	x–	*Cratena peregrina*	–	x
*Aplysina* sp.	x	x	*Flabellina sp.*	–	x
*Axinella cannabina*	x	x	*Pteria hirundo*	x	–
*Axinella polypoides*	–	x	*Felimare picta*	–	x
*Chondrilla nucula*	x–	x	*Peltodoris atromaculata*	–	x
*Chondrosia reniformis*	x	x	*Phyllidia flava*	–	x
*Cliona rhodensis*	x	x–	Anellids		
*Cliona celata*	x	x	*Protula tubularia*	x	x
*Cliona viridis*	x	x	*Serpula vermicularis*	x	x
*Crambe crambe*	x	x	*Hermodice carunculata*	–	x
*Cymbaxinella damicornis*	x	x	Echinoderms		
*Cymbaxinella verrucosa*	x	x	*Paracentrotus lividus*	–	x
*Dictyonella incisa*	–	x	*Sphaerechinus granularis*	–	x
*Dysidea avara*	x	x	*Echinaster sepositus*	–	x
*Dysidea fragilis*	–	x	*Marthasterias glacialis*	–	x
*Dysidea incrustans*	x	–	*Ophioderma longicauda*	–	x
*Geodia cydonium*	x	x	*Ophiotrix fragilis*	–	x
*Haliclona fulva*	x	x	*Holothuria polii*	–	x
*Haliclona mediterranea*	–	x	Ascidians		
*Haliclona mucosa*	x	–	*Clavelina lepadiformis*	x	-
*Hexadella racovitzai*	x	-	*Halocynthia papillosa*	x	x
*Ircinia oros*	x-	x	*Microcosmus sabatieri*	x	x
*Ircinia variabilis*	x	x			
*Leucosolenia variabilis*	x	-			
*Leucosolenia complicata*	x	–			
*Paraleucilla magna*	x	–			
*Oscarella lobularis*	x	–			
*Petrosia (Petrosia) ficiformis*	x	x			
*Phorbas tenacior*	x	x			
*Pleraplysilla spinifera*	x	x			
*Sarcotragus cf.spinosulus*	–	x			
*Sarcotragus foetidus*	x	–			
*Spirastrella cunactatrix*	x	x			
*Tethya* cf*. meloni*	–	x

**Figure 4:**
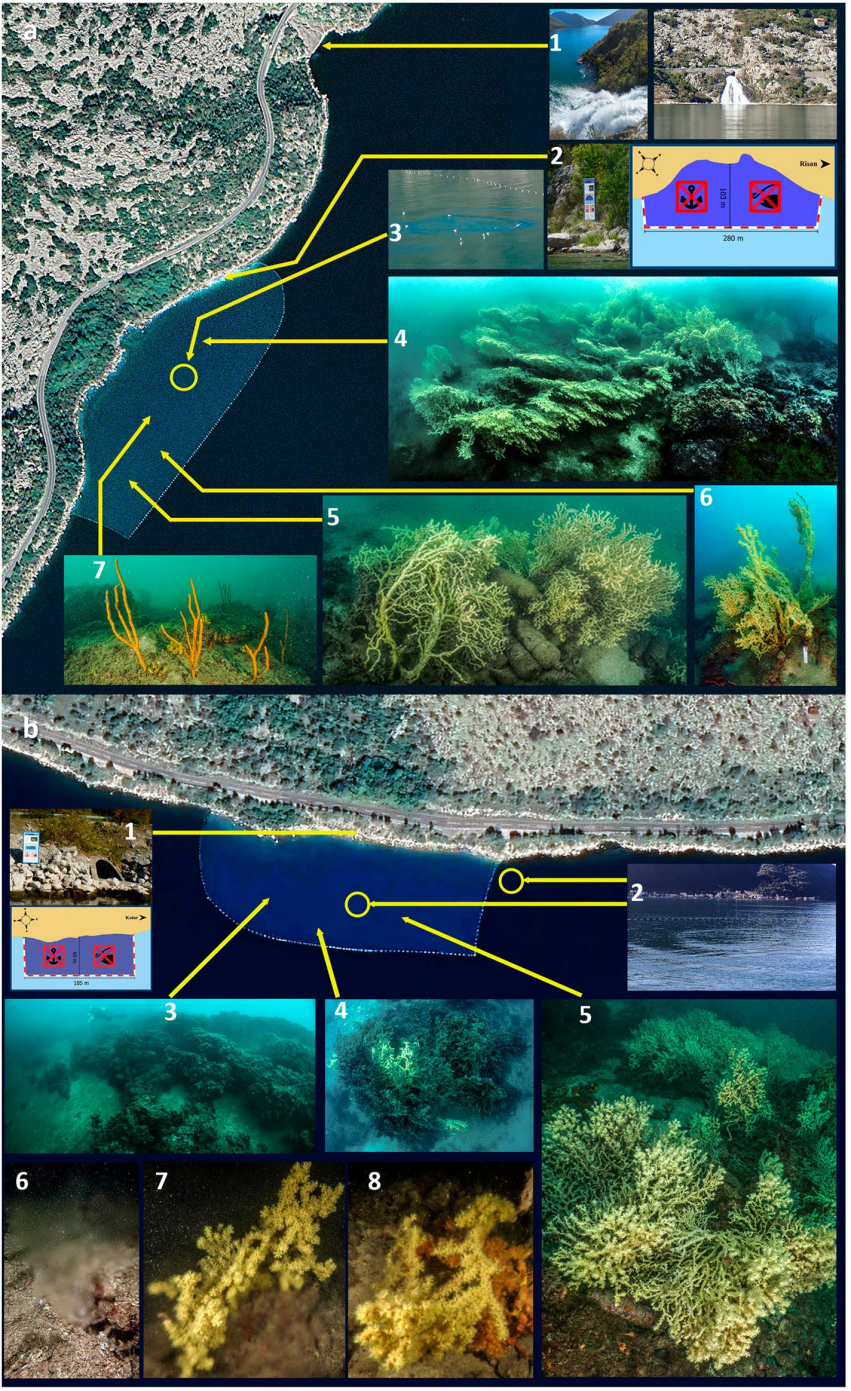
(**a**) The Sopot protected area. a1 the freshwaters outflows, 250 m northern of the protected area evidenced by a line of floating buoys; a2 sign on the coast indicating the protected area; a3 outgoing flow of freshwater (*vrulja*) visible from the surface; a4-5 the main *Savalia savaglia* patches of the area; a6 the higher recorded colonies in the patch; a7 *Axinella polypoides* facies, exclusive of this site. The Sopot aerial image is modified by Google Earth—Image © 2024 Maxar Technologies. (**b**) The Dražin vrt protected area. b1 position of the sign on the coast indicating the protected area; b2 positions of the *vrulja* present in the area; b3 *Cladocora caespitosa* reefs; b4-5 the deeper patches of *S. savaglia* where signs of necrosis were recorded; b6 image of the freshwater flowing from the detritic bottom; b7-8 *S. savaglia* colonies directly involved in the *vrulja* outflow. The aerial image of Drazin vrt is modified by Google Earth—Image © 2024 CNES Airbus.

At lower depths (< 10 m) *C. caespitosa* formed sparse small cushion-like colonies in association with emisciaphilic algae (*H. tuna*, *Peyssonnelia* spp.); moreover, *P. muellerae* and *P. americana mouchezii* built patchy reefs that exceed 1 m hight with a volume greater than 1 m^3^. Among the scleractinian reefs the bottom is widely detritic and populated by several species of sponge, particularly large, erected specimens of the genus *Axinella*: an *A. polypoides* facies was exclusively present in Sopot while *A. cannabina* was mainly recorded in Dražin vrt (Fig. 4). The layer 10–25 m is characterized by the *S. savaglia* forests together with very rare gorgonians (Figs. 3, 4).

### Occurrence and population structure of *Savalia savaglia*

In previous campaigns carried out between 2013 and 2023, it was possible to identify the locations where *S. savaglia* is currently established. Among the 107 visited sites, colonies of *S. savaglia* were found in only 5 (Fig. 1): they are Dražin vrt, Sopot, Strp, Sveti Djordje and Sveta Nedielja. The first 4 are in the innermost part of the bay, while the fifth is located in the Verige strait which connects the external areas to the internal ones. Ten colonies were observed in Strp, while in Sveti Djordje and Sveta Nedielja one colony for each site was recorded (unpublished data). At Sopot (Fig. 4a), *S. savaglia*, was distributed in a depth range of 8–17 m, both in sedimentary areas and, to a lesser extent, on bioconcretions and boulders, arranged both in three metric patches and sparse colonies. The main patch included 483 colonies and about 200 were counted in the other two. The real density was difficult to estimate because the zoanthid was arranged in crowded patches and colonies were frequently anastomosed on each other. The number of colonies counted is the result of an estimation that considers each identifiable fan as a colony.

The largest assemblage occupied an area of about 9.5 m^2^. The average height of the colonies was 48.5 ± 4.3 cm with a maximum of about 105 cm.

At Dražin vrt, (Fig. 4b) *S. savaglia* occurred between 9 and 28 m depth, both on detritic bottoms and rocks as large patches of several colonies. Here, 509 colonies were counted. The largest patch occupied an area of about 7.5 m^2^. The average height of the colonies was 43.4 ± 3.4 cm with a maximum of 85 cm.

The shape of the colonies was different probably according to different environmental conditions. In areas characterized by laminar water flow the colonies developed as completely planar (Fig. 4a4–6). On the contrary, around *vrulja*, where the current is mainly turbulent, colonies were three-dimensional, bush-like (Fig. 3b3–5), in particular as showed by specimens directly involved in the freshwater outflow (Fig. 4b6–8).

### Colony growth

**Figure 5:**
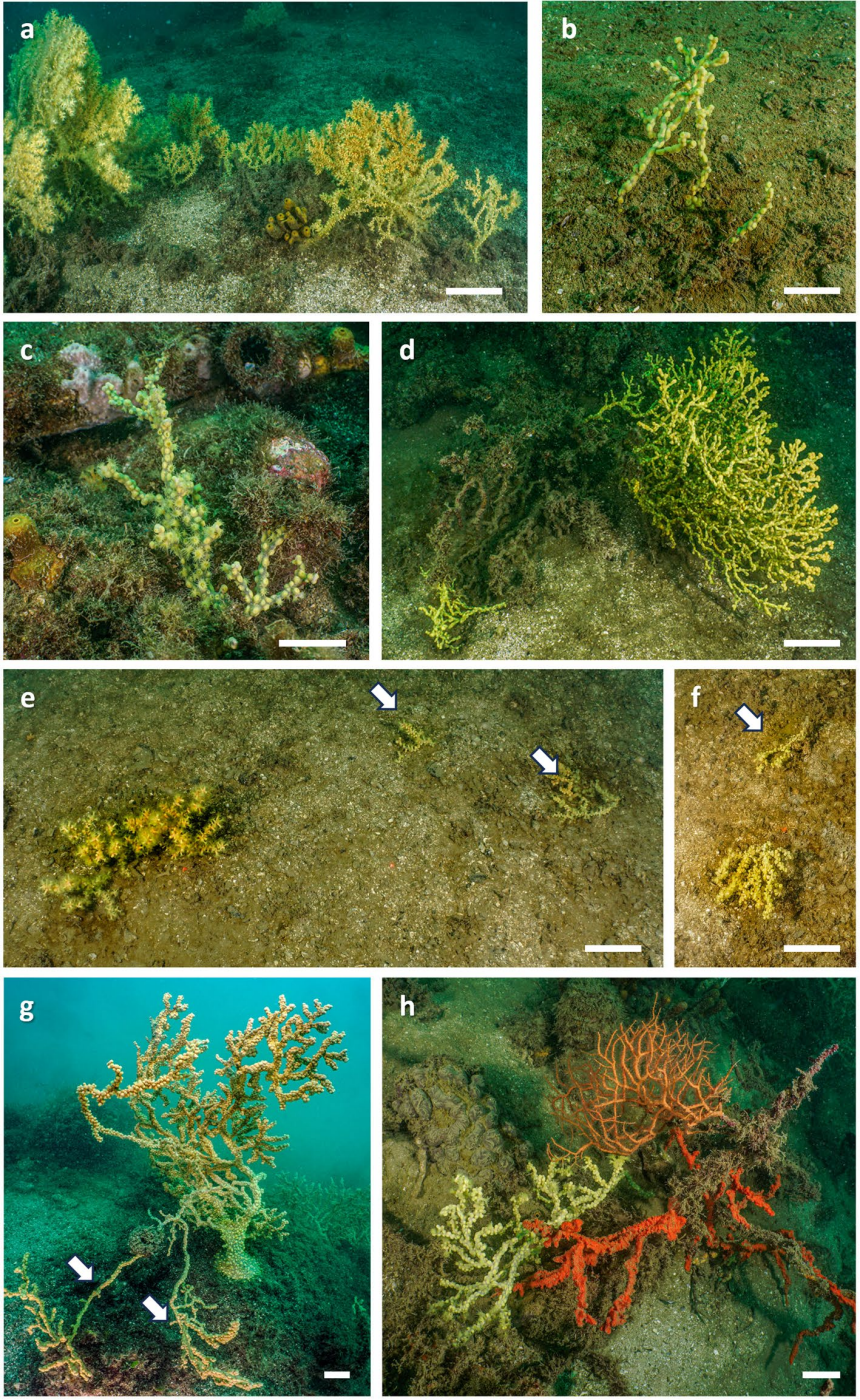
(**a)** occurrence of small colonies of *Savalia savaglia* together with larger ones; (**b**,**c**) small branched and monopodial specimens; (**d**–**f**) small colonies (white arrows) developed by asexual reproduction via fragmentation; (**g**) example of a large specimen with a large basal plate and lateral stolons; (**h**) a parasitic specimen of *S. savaglia* covering a *Leptogorgia sarmentosa* branch. Scale bars: 4 cm.

**Figure 6:**
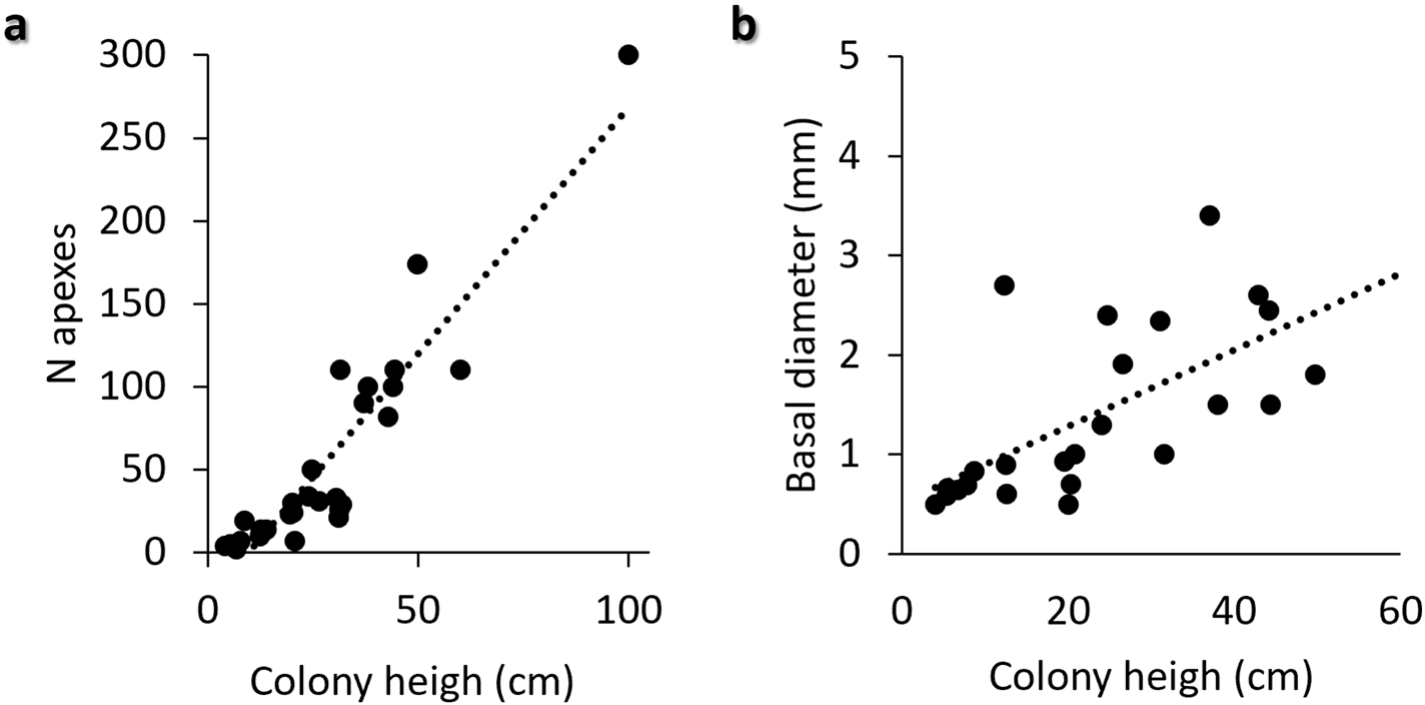
Linear relationship between the colony height and number of apexes (**a**), and the diameter of the stem (**b**).

Inside this dense population, we had the possibility to describe the growth of colonies. Several small colonies were regularly observed intermixed with the larger ones (Fig. 5a–d). The smallest recorded colonies were 4 cm high with a stem of about 0.3 cm wide. At this stage, they were sometimes monopodial (Fig. 5b) but little branched colonies with 2–5 apexes were frequently observed (Fig. 5b). During the following steps, the colonies linearly increased the number of apexes according to the colony height (r = 0.93; p<0.01) (Fig. 6a). A significant relation, although less strong (r = 0.64; p<0.01) was observed between the colony height and the basal diameter of the stem (Fig. 6b).

The recorded FD, on average 1.61 ± 0.04, remained unvaried among the size classes.

Living fragments of colonies of different sizes were frequently observed spread on the sea floor. In some cases, the observed small specimens were produced via asexual reproduction (Fig. 5d–f).

Sometimes, it was possible to observe, particularly in large specimens, the presence of very long unbranched offshoots that bent to get up to the bottom like strawberry runners. When these “stolons” reached the bottom, they started to form a basal plate from which new colonies were asexually produced (Fig. 5g).

In the present study area, the typical parasitic behaviour of the species was rarely observed: in fact, only six colonies, in direct contact with *L. sarmentosa*, showed colonisation of the gorgonian branches (Fig. 5h).

### Skeleton structure

**Figure 7:**
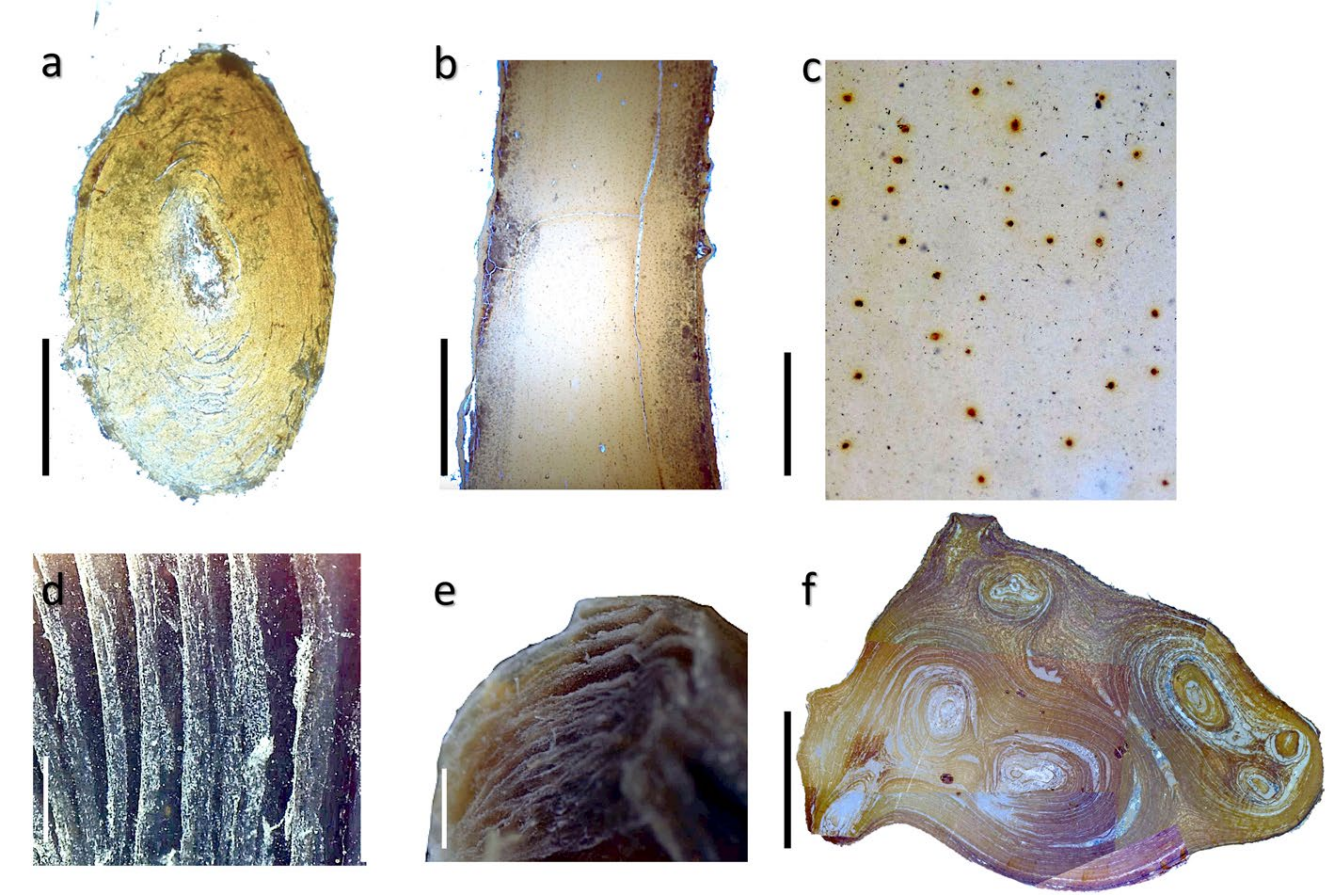
(**a**) thin sections of the basal part of the trunk of a specimen of *Savalia savaglia* without an evident gorgonian core; (**b**) longitudinal section of the skeleton showing its homogeneous aspect; (**c**) enlargement of the longitudinal section showing dense nodules spread in the skeleton; (**d,e**) the lamellar aspect of the organic skeleton after maceration in NaOH 2M at 45 °C for 5 days; (**f**) section of the trunk of a large specimen showing the presence of several stems enveloped by an external sheet of skeletal material. Scale bars: (**a**) 5 mm; (**b**) 0.5 mm; (**c**) 0.2 mm; (**d**) 0.1 mm; (**e**) 0.3 mm; (**f**) 1 cm.

The thin sections analysis of the basal part of the trunk of five specimens revealed a homogeneous structure of the skeleton without an evident gorgonin core, as generally shown in the parasitic specimens (Fig. 7a). The same homogeneous aspect was also visible by the longitudinal section (Fig. 7b). In these sections, dense nodules about 0.1 mm in diameter, until now never described, were visible spread in the matrix (Fig. 7c).

The partially dissolved portions of the skeleton highlighted the typical arrangement in concentric sheets from the periphery to the central portion of the trunk (Fig. 7d,e).

In a large specimen with a basal diameter of 4 cm wide, the skeleton was composed of many (up to five) single stems of different sizes (from 0.46 to 1.32 cm) each one with the characteristic concentric growth pattern; the more external portion of the section was formed by concentric sheets that enveloped the entire bundle of stems (Fig. 7f).

### Damages, epibiosis and necrosis

**Figure 8:**
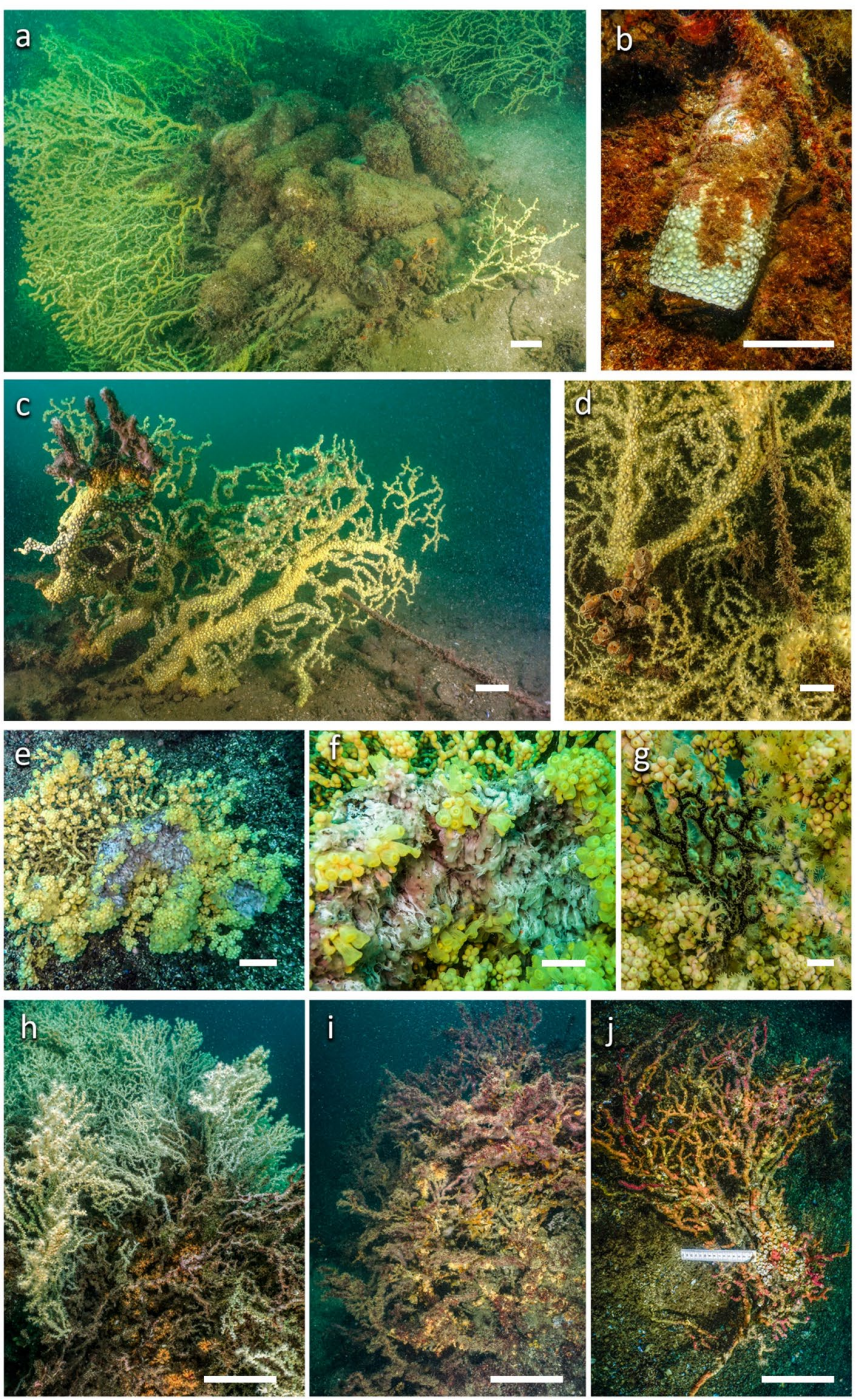
Examples of the anthropogenic pressures and diseases affecting *Savalia savaglia***: **(**a**) a huge amount of marine litter bending or completely covering the colonies; (**b**) a plastic bottle covered by an encrusting colony; (**c**) a large colony entangled in a nylon line overgrown by the sponge *Dysidea* sp.; (**d**) another entangled colony overgrown by *Aplysina* sp.; (**e**,**f**) a colony affected by necrosis resulting in a mass of mucous white–gray substance with the apical portions of the branches re-arranged in spherical inflated structure; (**g**) naked branched and (**h-i**) a large dead portion of colony; (**j**) a completely dead colony covered by several epibionts. Scale bars: (**a**–**e**) 6 cm; (**f**,**g**) 1 cm; (**h**–**j**) 15 cm.

In the area of Boka Kotorska Bay, the sea floor was heavily impacted by a large amount of urban waste: the marine litter (nets, lines, glass and plastic bottles, cans, pots, tires, casks and large metallic debris) resulted particularly widespread and abundant in both the investigated sites and frequently involving the patches of *S. savaglia*, bending or covering the colonies (Fig. 8a). The density of wastes was 0.18 objects m^−2^ at Dražin vrt and 0.14 objects m^−2^ at Sopot protected area. In some cases, *S. savaglia* was able to overgrow plastic debris producing laminar colonies (Fig. 8b).

The epibiosis on the colonies was rare at Sopot while at Dražin vrt it was more frequent. In these cases, epibionts were constituted by algae, the octocoral *Sarcodictyon catenatum* and sometimes large sponges, in particular *Aplysina* sp., *Dysidea avara, Petrosia* *(Petrosia)* *ficiformis* (Poiret, 1789) (Fig. 8c,d). Sometimes, partially or completely dead colonies covered by epibionts were recorded still in place.

Moreover, the impact due to fishing gears was widely observed with lost lines entangled in the branches of the colonies (Fig. 8c,d); in some cases, it was possible to record the growth of the *S. savaglia* polyps on the nylon lines. The line entanglement frequently resulted in cutting off some branches up to the extirpation of colonies.

Signs of necrosis were observed as masses of mucous white–gray substance involving portions of the branches (Fig. 8e). In some colonies, partially affected by necrosis, polyps were re-arranged producing a spherical inflated structure at the apexes of the branches (Fig. 8f). The necrosis can evolve into a naked skeletal portion and involve large parts, up to the entire colony (Fig. 8g–i).

## Discussion

The present study provides a description of the benthic communities associated with cold freshwater springs at shallow depths in Boka Kotorska Bay. In this area, the diversity is high, particularly regarding massive or branching sponges. This high richness could be tentatively related to the putative high concentration of SiO_4_ in the waters emitted by the springs. Studies conducted in similar situations along the Croatian coasts indicate in the *vruljas*’ plume a SiO_4_ concentration of about one order of magnitude higher than in the surrounding marine waters^43^. In Indonesian waters, rich sponge assemblages grow in close association with thermal outflows with a high silica concentration^44^. Also, the great development of scleractinian bioconstructions could be hypothetically related to an increase in carbonate availability provided by *vrulja* outflows^43,45^.

The most impressive element of this community is the *Savalia savaglia* population, representing a unique Mediterranean example of the gold coral forest. The main two explored sites account for more than 500 colonies each, concentrated in the proximity of the main submarine freshwater springs. Along the Montenegrin coast, *S. savaglia* is a very rare species. Unpublished surveys conducted by diving for more than 25 years by one of us (VM) stated that this zoanthid was never found out of the Boka Kotorska Bay, while in the bay, out of more than 100 dive locations, it was found at only 5 (Fig. 1). In Sv. Nedjelja, Sv. Đorđe island and Strp it was recorded only as scattered colonies while the only two sites characterised by a *S. savaglia* forest are Sopot and Dražin vrt. So, the presence of freshwater springs arising directly from the bottom seems to play an important role in the development and shaping of *S. savaglia* population. The first diving exploration of the area observed that the release of cold freshwater, the strong mixing of the water masses and the relative turbidity of the places have favored the growth of a *S. savaglia* forest, with colonies of metric height^29^.

The high quantity of freshwater that flows into the bay mostly in Winter and Spring, coming both from superficial coastal runoffs and from upwelling from submerged springs (*vrulja*) has the consequence of a strong reduction in light penetration due to clouding, which explains the observed vertical compression of the planes and the minimum settlement depth ever recorded for the species (8 m). Moreover, the action of *vrulja* produces a substantial upwelling of organic matter from the sediments over a long span of time across the year determining this impressive flourishment of the species.

A new finding is the ability of *S. savaglia* to tolerate very low salinity levels for extended periods. In fact, we have documented colonies directly placed inside the freshwater flow (Fig. 4b). Although salinity is known to be one of the limiting factors in anthozoan development, tolerance towards low levels of this parameter was recorded in different species^45–49^ and particularly among zoanthids^50^.

Recently, Pulido Mantas et al.^20^ updated the distribution of the species in the Mediterranean Sea and the Eastern Atlantic Ocean considering about 70 localities where it occurs. In more than 90% of the explored sites *S. savaglia* was present with less than 5 colonies, clearly demonstrating that the species is rare in the greater part of its distribution area. Beyond the Boka Kotorska population, the only important exception is that of the Canary Archipelago, where it forms a forest with more than 1000 colonies in relatively shallow waters (27–70 m)^51^. However, the structure of this population, composed by well-spaced colonies, is very different from the large dense patches of anastomosed colonies found in Montenegro. Unfortunately, the environmental conditions of these two localities, one oceanic and one coastal Mediterranean, are completely different and do not allow us to understand the drivers able to promote the formation of wide populations.

A possible solution could be found in the peculiar way of reproduction and growth of these populations. In the Boka Kotorska Bay, it is much more than likely that *S. savaglia* colonies generally have no parasitic behaviour. This seems demonstrated by different aspects. Firstly, at the sites where *S. savaglia* is present, gorgonians are virtually absent or very rare. Secondly, the sections of the basal portion of the stem of the colonies do not reveal the putative inner skeleton of the covered gorgonians. Finally, in this population, it is common to find very small colonies, less than 5 cm high, in some cases only formed by a creeping plate. It is clear that these small specimens cannot be parasites. This bulk of evidence induces us to consider that this population is able to grow on its own skeleton without the need for the support of living gorgonians. The same situation was observed for the Canary Arch. population^51^. It is therefore very probable that when *S. savaglia* lives without following a parasitic strategy, it can form very large aggregations.

The use of ddRAD-Seq^21^ genotyping on individuals collected from the Tyrrhenian Sea, Adriatic Sea (comprising the population of the Boka Kotorska Bay) and the Eastern Atlantic highlighted the presence of three main genetic clusters, two in the Tyrrhenian Sea and one comprising the two Adriatic populations and the Portuguese one. New research will be necessary to establish if the specimens of this peculiar genetic cluster are more related to a non-parasitic lifestyle.

In the same paper, it was stated that the strong linkage imbalance recorded across loci and the detection of clonal individuals in shallow populations suggest that the *S. savaglia* populations adopt asexual reproduction as the main reproductive strategy^21^. During our survey, we recorded several hints that support this hypothesis as the numerous portions of branches recorded alive on the seafloor and the small, settled colonies. The evidence that, on about one thousand colonies studied we had not observed specimens lesser than 4 cm in height strongly suggests that these small colonies derived by the re-attachment of the fragments on the new substrate.

Anthozoans are known to commonly reproduce by fragmentation^52–55^, with the separation from the coral colony of a living portion that, after re-attachment, produces a new colony. Recently, Coppari et al.^56^ reported the observation of an extensive event of fragmentation in the Mediterranean black coral species *Antipathella subpinnata* (Ellis & Solander, 1786) (Antipatharia: Myriopathidae) in rearing conditions. Once detached, fragments lose their polarity and new anchorages are rapidly created with polyps and cnidocysts participating in the adhesion phases.

Another evidence of asexual reproduction was the record of stolonisation phenomena arising from old colonies and producing new ones. Growth via stolonisation in exacorals was rarely observed and it is suggestive that one of the few available examples was observed in zoanthids where *Zoanthus sociatus* (Ellis, 1768) has a stoloniferous growth form exhibiting both runner and sheet morphologies^57^.

The record of colonies of different sizes allowed, for the first time, to describe the proper growth pattern of this species. In fact, when the gold coral parasites a gorgonian, the type of growth is constrained by the shape of the host. We have found the fractal dimension of *S. savaglia* stable across different size classes (1.31–1.89; on average 1.61 ± 0.04). This suggests the species early achieved its optimal branching pattern that remained stable with growth. This data is in agreement with the linear relationship between colony height and apexes number. A very similar fractal dimension (D = 1.58) was recorded for the gorgonian *Leptogorgia sarmentosa*^58,59^.

The colony size, particularly the stem diameter, seemed to be related to the coalescence of several colonies settled in close groups. It is evident that, after a period of growth, the different colonies are enveloped by a unique layer of coenenchyme that, in turn, produces a common layer of organic skeleton. The formation of chimeric colonies has been widely studied mainly in tropical scleractinians^60,61^. Under experimental conditions, 1.5-fold more larvae of *Acropora (Acropora) millepora* (Ehrenberg, 1834) settled in aggregations than solitarily and the resulting chimeric colonies persisted for a long time. Data showed that chimeric colonies grow faster compared with solitary juveniles indicating chimerism as an important strategy for maximizing survival of vulnerable early life-history stages of corals^62^.

Despite the high naturalistic value of this unique *S. savaglia* population, the areas where the forests settle were recorded as heavily impacted by urban pollution. Due to their unique benthic assemblages, the sites of Sopot and Dražin vrt, have been placed under preventive protection in July 2021. Although cleaning activities were already conducted in 2020 and 2021^63^, the situation remains critical especially at Sopot, with the high quantity of plastic bottles accumulated, which heavily impacts *S. savaglia* colonies. The pending governmental declaration of permanent protection is highly awaited according to the National Law on Nature Protection^64^, in order to provide high-quality management. Although *S. savaglia* demonstrated a high degree of resilience, the requalification of the area, currently 4.1 ha in total (0.3% of the total area of Boka Kotorska) should be expanded to other sites where the species is present (i.e. Strp), and to buffer areas. The institution of a Marine Protected Area, with different levels of protection, would be able to preserve the richness and rarity of these whole high value of the *vrulja*’s communities.

## Data Availability

The authors confirm that the data supporting the findings of this study are available within the article. The photographic dataset collected and analyzed during the present study is available from the corresponding author upon request.
